# Tumor resting-state fMRI connectivity to extralesional brain is associated with cognitive performance in glioma patients

**DOI:** 10.1016/j.bas.2025.104202

**Published:** 2025-02-03

**Authors:** Chuh-Hyoun Na, Hans Clusmann, Martin Wiesmann, Kerstin Jütten, Verena Mainz

**Affiliations:** a Department of Neurosurgery, RWTH Aachen University, Pauwelsstraße 30, 52074, Aachen, Germany; b Center for Integrated Oncology Aachen Bonn Cologne Duesseldorf (CIO ABCD); c Department of Diagnostic and Interventional Neuroradiology, RWTH Aachen University, Pauwelsstraße 30, 52074, Aachen, Germany; d Institute of Medical Psychology and Medical Sociology, RWTH Aachen University, Pauwelsstraße 19, 52074, Aachen, Germany

**Keywords:** Resting-state fMRI, Cognitive outcome, Glioma, Functional connectivity, Connectome

## Abstract

**Introduction:**

Functional coupling of the tumor to extralesional brain areas and the pretherapeutic cognitive performance status have each independently been identified as prognostically relevant in glioma patients. It is however unclear, whether tumor-connectivity correlates with cognitive performance or the cognitive outcome.

**Research question:**

To investigate potential associations between pre- and postoperative resting-state fMRI connectivity (FC) and cognitive functions in glioma patients compared to healthy controls.

**Material and methods:**

18 patients and 18 age-matched, healthy controls underwent resting-state fMRI and neuropsychological testing pre- and 4.5 months (mean) postoperatively. FC of the tumor to extralesional brain (Tu-EL) was determined, as well as FC of extralesional brain (EL) and the contralesional hemisphere (conEL). Groups were compared with regard to behavioral and FC measures.

**Results:**

Patients showed deficits in all cognitive domains tested. While postoperative performance tended to be worse, deterioration was not statistically significant between timepoints. EL FC did not differ between groups, but conEL FC (p < .045) was increased in patients as compared to controls. Tu-EL FC was significantly associated with worse attention performance (p < .001), and, by trend (p < .058), with worse attentional outcome in patients.

**Discussion and conclusion:**

Intrinsic functional coupling to the rest of the brain was associated with worse cognitive performance and might relate to pathological tumor-neuron interaction on the macroscale, reflecting the invasive nature of diffusely infiltrating glioma. Deepening our understanding of FC measures at the connectomic level in the context of cancer neuroscience may aid in identifying neurophysiological correlates of cognitive impairment and in prognosticating cognitive outcome in glioma patients.

## Highlights

• Glioma patients show cognitive deficits pre- and postoperatively.
• Patients show hyperconnectivity in the contralesional hemisphere.
• A high tumor-brain functional connectivity relates to worse attentional performance.
• Tumor-brain functional connectivity may indicate postoperative attentional outcome.
• Dysfunctional tumor coupling may reflect tumor-neural interaction on the macroscale.

## 1 Introduction

Glioma patients frequently suffer from neurocognitive deficits (Habets et al., 2014; Noll et al., 2015) which significantly impact quality of life and the capability for socioaffective integration of the individual. Estimating the lesion impact on cognitive functions and postoperative cognitive outcome in glioma patients is however difficult in view of the diffusely infiltrating tumor nature and the spatiotemporally widely distributed network representations of higher order functions. Compared to motor or language functions, neurophysiological correlates and anatomical boundaries of eloquent brain regions regarding cognitive functions are less well defined, and pre- or intraoperative mapping techniques for cognitive functions are not commonly established. Surgical intervention may improve or deteriorate cognitive functions, but the individual cognitive outcome is difficult to predict.

Global connectivity alterations have been associated with neurocognitive functions and lesion momentum of prognostically differing glioma types (Derks et al., 2017, 2019; Kesler et al., 2017; Stoecklein et al., 2020), and have even been linked to survival outcome in malignant gliomas (Lamichhane et al., 2021; Liu et al., 2019). Moreover, the pretherapeutic cognitive performance status has been identified as an independent predictor of glioma patients’ overall survival (van Kessel et al., 2021; Meyers et al., 2000). Cognitive function and functional connectivity (FC) measures on the connectomic level thus may inherently provide information about the degree of systemic cerebral involvement and the individual prognosis in glioma patients, even in lack of histological or molecularpathological specifications prior to surgical intervention.

Longitudinal studies investigating resting-state fMRI connectivity alterations related to postoperative cognitive outcome in glioma patients are however rare. Studies on whether preoperative FC-based measures might be informative for cognitive outcome predictions in surgical intervention are even rarer, but are of high interest, given that cognitive outcome was not related to presurgical tumor characteristics or extent of resection in previous studies (Habets et al., 2014). An MEG study investigating resting-state FC of glioma lesions in eloquent/perieloquent tumor locations showed decreases in FC of the tumor region to the remaining brain to have a negative predictive value of 100% for the absence of sensory-motor or language responses during intraoperative electrical stimulation (IES). Increases in FC were described to have a positive predictive value of 64% for eloquent regions during IES mapping (Martino et al., 2011). Accordingly, a consecutive MEG study by Tarapore et al. (2012) showed, that patients with decreased FC of the tumor region to whole-brain (relative to the connectivity of the homologuous region of the contralesional hemisphere) had a lower rate of postoperative neurological deficits, while those with tumor lesions with increased FC were associated with a higher rate of postoperative neurological deficits. Whether MRI resting-state FC based on synchronized blood-oxygen-level-dependant (BOLD) low frequency oscillations of the tumor to the rest of the brain might as well be predictive for the cognitive outcome has however not been clarified yet.

In a first exploratory approach, we therefore longitudinally investigated cognitive performance and resting-state fMRI connectivity pre- and postoperatively in glioma patients compared to controls. We hypothesized that cognitive performance and cognitive outcome would relate to FC of the tumor and of extralesional brain areas.

## 2 Materials and methods

### 2.1 Participants

18 patients with unilateral and histopathologically proven cerebral glioma according to the revised WHO tumor classification of 2016 (Louis et al., 2016) (12 left-hemispheric supratentorial tumors; 11 with Isocitrat-Dehydrogenase [IDH]1 mutation; 17 naíve to tumor-specific treatment; mean age: 46 ± 4 years; 11 males; 16 right-handed; time interval between assessments: 4.5 ± 1.8 months) and 18 age-matched, healthy controls (mean age: 47 ± 1 years; 12 males; 16 right-handed; time interval between assessments: 3 months) were included in the study and comprised a follow-up assessment. For congruency in writing and for the purpose of readability, the control group's initial and follow-up assessment time points will be referred to as pre- and post-operative assessment, in equivalence to those of the patient group.

Only patients ≥18 and < 80 years of age with a Karnofsky index of ≥70 were included in the study. Please note that the study sample partially overlaps to study cohorts previously published (Jütten et al., 2020; Jütten et al., 2019; Jütten et al., 2021). Patients’ demographics and tumor characteristics can be found in Table 1. All participants gave informed written consent. The study was approved by the ethics committee of the Medical Faculty of the RWTH Aachen University (EK294-15, date of approval: 22.03.2016) and conducted in accordance with the standards of Good Clinical Practice and the Declaration of Helsinki.

**Table 1:** Clinical description and demographics of included patients.

Patients	IDH-mutation	Diagnosis	Grade	Location	Side	Volume (in cm³)	Age (years)	Education (years)^c^	Radiotherapy	Chemotherapy
1	y	Astrocytoma	II	Insular	r	30	30-35	13	n	n
2	y	Astrocytoma	II	Insular	l	52	30-35	15	y	y
3	y	Astrocytoma	II	Frontal	l	56	26-30	16	n	n
4	y	Oligodendroglioma^a^	II	Frontal	l	73	26-30	13	y	y
5	y	Astrocytoma	II	Parietal	l	86	56-60	18	y	y
6	y	Anaplastic astrocytoma	III	Parietal	r	30	56-60	9	y	y
7	y	Anaplastic astrocytoma	III	Frontal	l	33	50-55	13	y	n
8	y	Anaplastic oligodendro-glioma^a^	III	Frontal	l	53	50-55	15	y	y
9	y	Anaplastic astrocytoma	III	Parietal	l	91	20-25	13	y	y
10	y	Anaplastic oligodendro-glioma^a^	III	Frontal	r	100	30-35	18	n	n
11	y	Anaplastic oligodendro-glioma^a^	III	Frontal	r	158	30-35	13	y	y
12	n	DNT	I	Temporo-parietal	l	18	40-45	15	n	n
13	n	Glioblastoma multiforme^b^	IV	Frontal	l	11	76-80	13	n	n
14	n	Glioblastoma multiforme	IV	Temporo-parietal	l	13	56-60	10	y	y
15	n	Glioblastoma multiforme	IV	Temporo-parietal-occipital	l	14	60-65	15	n	y
16	n	Glioblastoma multiforme	IV	Frontal	r	40	50-55	12	y	y
17	n	Glioblastoma multiforme	IV	Occipital	l	54	50-55	13	y	y
18	n	Glioblastoma multiforme	IV	Frontal	r	69	66-70	12	y	y

*Note*. IDH=isocitrate-dehydrogenase, y=yes, n=no, l=left, r=right.

a Patients with codeletion of chromosome arms 1p and 19q.

b Recurrent tumor with preceding tumor resection and adjuvant radiochemotherapy.

c Years of education were computed by the sum of years spent for school career and further training/study.

### 2.2 Neuropsychological assessment

Participants underwent a standardized neuropsychological examination, which has been described in detail previously (Jütten et al., 2020; Jütten et al., 2019). Examination included the Verbal Learning and Memory Test (VLMT) (Helmstaedter et al., 2001), the Attention Network Test (ANT) (Fan et al., 2002), as well as the Trail-Making-Test (TMT) (Reitan and Wolfson, 2004). The VLMT assesses verbal learning and recall. Analyses focus on learning, consolidation and recognition scores. The ANT is used to examine different attentional systems. Analyses focus on overall alertness by investigating the quotient of mean reaction times of correct trials and number of correct responses. To assess executive functioning, the TMT was used. The difference in completion time recorded separately for each of two test parts (TMT-A, TMT-B) reflects cognitive flexibility.

### 2.3 MRI data acquisition

MRI examination was applied using a 3T Siemens Prisma MRI scanner equipped with a standard 20-channel head coil. The scanning protocol is described in previous studies (Jütten et al., 2020; Jütten et al., 2019; Jütten et al., 2021), and comprised the following pulse sequences: First, a sagittal 3D T1 magnetization-prepared rapid acquisition gradient echo (MPRAGE) sequence was acquired (repetition time [TR] = 2,300ms, echo time [TE] = 2.01ms, 176 slices with a slice thickness of 1 mm, flip angle = 9°, field of view [FoV] = 256 mm, voxel size = 1 mm isotropic, and 256 × 256 matrix). In addition, a fluid attenuation inversion recovery (FLAIR) sequence was applied (TR = 4,800ms, TE = 304.0ms, number of slices = 160 with 1 mm slice thickness, FoV m s250mm, and 1 mm isotropic voxel resolution). For tumor identification purposes, a contrast-enhanced, T1-weighted turbo inversion recovery magnitude (TIRM) dark-fluid sequence was acquired (TR = 2,200ms, TE = 32ms, slice thickness = 3 mm, flip angle = 150°, FOV = 230 mm, voxel size = 0.9 × 0.9 × 3.0 mm3, matrix = 256 × 256) as well as a T2-weighted TIRM darkfluid scan (TR = 9,000ms, TE = 79ms, slice thickness = 3 mm, flip angle = 150°, FOV = 230 mm, voxel size = 0.9 × 0.9 × 3 mm3, matrix = 256 × 256). In addition, RS-fMRI was implemented using echo planar imaging (EPI), including 300 whole-brain functional volumes, TR=2200 ms, TE=30 ms, number of slices=36 with 3.1 mm slice thickness, flip angle=90°, and FoV=200 mm.

### 2.4 Preprocessing

Functional preprocessing was performed using SPM12 (Friston et al., 2007; Penny et al., 2006) as implemented in Matlab 9.3 (Matlab 2017b. Version 9, 2017). A detailed description of the image preprocessing protocol can be found in previous studie (Jütten et al., 2020; Jütten et al., 2019; Jütten et al., 2021), and included semi-automatic tumor lesion segmentation using the ITK-SNAP software 3.6.0 (Yushkevich et al., 2006) Preoperative masking included perifocal T1 hypo- and T2-FLAIR hyperintensities for gliomas grade I-III, as well as T1 hypointensities, necrosis and contrast-enhancing tumor for glioblastomas, creating a binary tumor mask. After tumor resection, the resection hole was masked, creating a binary resection mask. In addition, a combined lesion mask was created, adding pre- and postoperative lesions. This pre-post lesional mask was used on final preprocessed functional images, as described below. Functional images were realigned to the mean functional volume, unwarped and coregistered to the structural T1-weighted image. Structural and functional images were normalized (including the binary tumor mask resp. resection mask in case of patients’ data), and functional images were smoothed with a 5 mm FWHM Gaussian kernel.

**Fig. 1:**
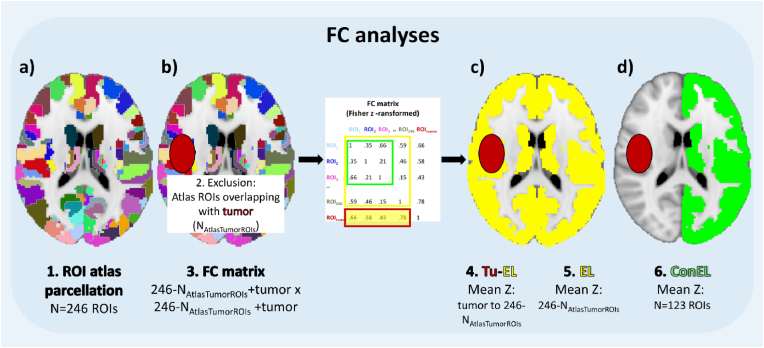
Schematic view of analyzed volumes. Fig. 1 Functional connectivity (FC) analysis workflow. Functional volumes were parcellated into a set of 246 predefined anatomical brain regions of interest (ROI, a). Patients' lesion masks (red circle) were used to exclude all atlas regions that cover tumor tissue (b). The remaining ROIs were used to create a whole-brain mask, building a FC matrix that included Fisher z-transformed correlations between all remaining atlas ROIs as well as the tumor ROI (in case of patients' data). Patients' preoperative tumor-to-extralesional (Tu–EL, red-yellow) FC was computed, as well as preoperative and postoperative extralesional (EL, yellow) FC in patients and controls (c). In addition, patients' pre- and postoperative FC of the contralesional hemisphere (conEL, green) was computed (d). For controls, contralesional FC was represented by the mean value of left- and right-hemispheric FC.

For FC analyses, movement related time series were regressed out with ICA-AROMA (Pruim et al., 2015), and grand-mean scaling (mean-based intensity normalization), high-pass filtering (>0.01 Hz) as well as slice timing correction were applied. Then, final preprocessed functional images were masked with the pre-post combined lesional mask in order to ensure intraindividually constant extralesional whole-brain volumes for longitudinal analyses. To reduce computational burden, functional volumes were parcellated into a set of 246 predefined anatomical brain regions using the Brainnetome atlas for whole-brain analyses (Fig. 1a). A list of included left- and right-hemispheric brain regions can be found here (https://atlas.brainnetome.org/download.html).

### 2.5 FC analyses

The analysis approach and applied methods are shown in Fig. 1. To investigate tumor-related FC to the rest of the cerebrum, tumor-to-extralesional-brain FC (Tu-EL) was computed. To do so, first, lesion masks were used to exclude all parcellated atlas ROIs that cover tumor tissue (N_AtlasTumorROIs_, Fig. 1b): Individual subjects' time-courses were extracted from the tumor as well as from each of the remaining parcellated atlas regions and the mean time-series of each ROI was computed. All ROIs' mean time-series were cross-correlated within each subject, resulting in a 246-N_AtlasTumorROIs_ + Tumor x 246-N_AtlasTumorROIs_ + Tumor FC matrix. For controls, cross-correlations resulted in a 246x246 FC matrix. Then, correlations were Fisher z-transformed, and Tu-EL FC was assessed by computing the correlation between (the mean z-value of) the tumor and all 246-N_AtlasTumorROIs_ atlas ROIs (Fig. 1c). To determine extralesional FC (EL), the mean z-value over all 246-N_AtlasTumorROIs_ atlas ROIs was computed likewise. Tu-EL FC, as well as pre- and postoperative EL FC was analyzed. In addition, extralesional FC of the contralesional hemisphere (conEL) FC was computed to account for FC alterations in tumor-far brain regions (Fig. 1d). As left- and right-hemispheric FC in controls did not differ for initial or follow-up EL (F(2,15)=.208; p=.814), the mean of left- and right-hemispheric FC was computed and used as baseline (“healthy”) that patients’ connectivity was compared with, in order to indicate an up- or downregulation of FC in patients.

### 2.6 Statistics

All statistical analyses were performed with SPSS 27 (IBM SPSS Statistics for Windows). Data measures deviating more than 1.5 standard deviations from the group-specific mean were regarded as outliers and corrected for by being replaced by the group-specific mean + - 1.5 standard deviations on that respective variable. This was the case for patients’ behavioral measures only, namely for preoperative alertness (one patient) as well as for postoperative verbal consolidation (two patients) and alterness (two patients). All significances were tested two-sided and Bonferroni-corrected.

#### 2.6.1 Initial and follow-up behavioral performance

To investigate differences in performance between groups at the initial and at follow-up, a Repeated-Measures Analysis of Covariance (ANCOVA) was applied, including initial and follow-up behavioral measures as within-subject (learning, consolidation, recognition, alertness, and cognitive flexibility), group (patients and controls) as between-subject variable, and age as covariate.

#### 2.6.2 Initial and follow-up FC

Differences in initial and follow-up EL FC between groups were analyzed using a Repeated-Measures ANCOVA, including initial and follow-up EL FC as within-subject, group (patients and controls) as between-subject variables, and age as covariate. Similarly, contralesional EL FC was compared in a second Repeated-Measures ANCOVA, including initial and follow-up conEL FC as within-subject, group (patients and controls) as between-subject variables, and age as covariate.

#### 2.6.3 Correlations between FC and behavioral performance

In controls, associations between initial and follow-up EL, conEL FC and behavioral performance were tested by Pearson's partial correlation analyses, accounting for age.

The relationship between patients' FC measures and behavioral performance before and after resection was tested using Pearson's partial correlation analyses, controlling for effects of age and tumor volume. Tested variables included initial and follow-up EL, conEL FC and behavioral performance, as well as preoperative Tu-EL FC.

All correlation analyses were tested two-sided with a significance level of p < .05 and Bonferroni-corrected for multiple testing, adjusted *p*=.003 (patients), *p*=.004 (controls).

## 3 Results

### 3.1 Initial and follow-up behavioral performance

**Fig. 2:**
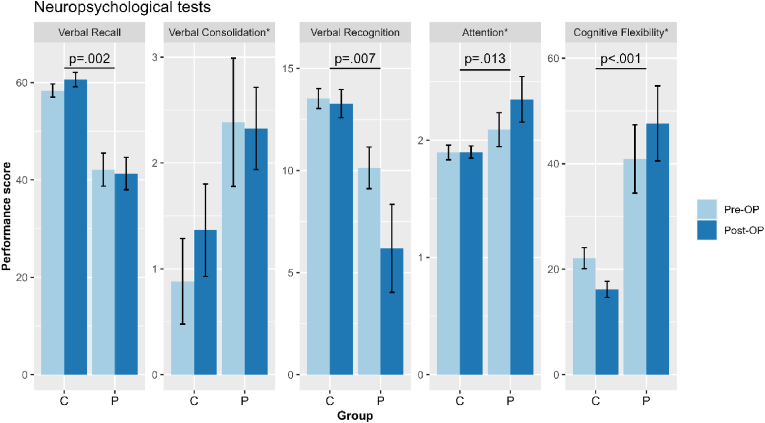
Behavioral data pre- and postoperatively Fig. 2 Neuropsychological performance differences between controls (C) and patients (P), with preoperative (pre-OP) and postoperative (post-OP) performance scores visualized in light and dark blue, respectively. Higher scores indicate better performance with regard to verbal recall and verbal recognition (number of correct words), whereas higher scores indicate worse performance in verbal consolidation (number of forgotten words), attention (quotient), and cognitive flexibility (reaction time).

Repeated-Measures results showed a significant main effect of group (*F*(5,17)=4.607, *p*=.008), indicating that patients revealed worse performance scores than controls in recall (*F*(1,21)=12.795, *p*=.002), recognition (*F*(1,21)=9.050, *p*=.007), alertness (*F*(1,21)=7.427, *p*=.013), and cognitive flexibility (*F*(1,21)=20.799, *p*=.000). The main effect of time of assessment, indicating the difference between initial and follow-up examination, was not significant, neither was the interaction of group by assessment time. For detailed results, see Fig. 2.

### 3.2 Initial and follow-up EL FC

Comparing initial and follow-up EL FC between patients and controls, results revealed a trend towards a main effect of assessment time (*F*(1,32)=3.455, *p*=.072), indicating a decrease in EL FC from initial to follow-up. Groups did not significantly differ in EL FC, as this main effect was not significant, as was the case for the interaction of group by assessment time.

**Fig. 3:**
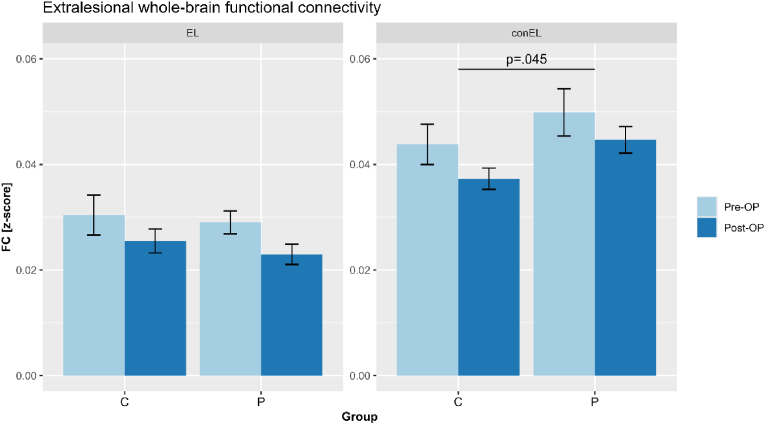
Functional connectivity of extralesional brain regions Fig. 3 Differences in extralesional (EL) and contralesional (conEL) functional connectivity (FC) between controls (C) and patients (P) are visualized, with preoperative (pre-OP) and postoperative (post-OP) z-scores shown in light and dark blue, respectively.

Significant group differences were found in conEL FC (*F*(1,33)=4.337, *p*=.045), indicating an upregulation of conEL FC in patients compared to controls. Neither initial and follow-up EL FC assessment, nor the interaction of group by time of assessment did reach significance, as revealed by the Repeated-Measures analysis, see Fig. 3.

### 3.3 Correlations between FC and cognitive outcome

In controls, partial correlation results did not reveal any significances, neither for correlations between FC and initial and follow-up behavioral performance.

**Fig. 4:**
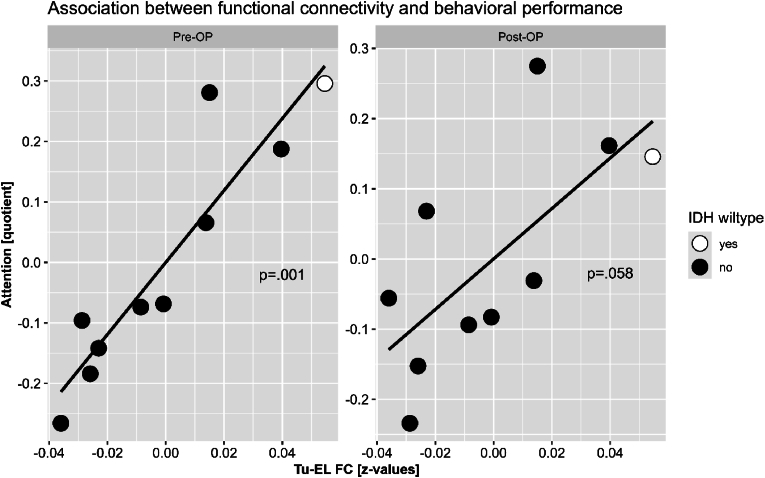
Correlation of Tumor BOLD connectivity to attentional performance Fig. 4 Partial correlation between patients' preoperative (pre-OP) as well as postoperative (post-OP) attention performance scores and tumor to extralesional (Tu-EL) functional connectivity (FC). Higher performance scores indicate worse performance in attention. Patients with Isocitrat-Dehydrogenase (IDH) 1 mutation are visualized as filled, black circles, and IDH wildtype patients in white. This study included a total of 7 patients with IDH wild-type gliomas, of which 6 patients could however only be tested either pre- or postoperatively, so that only complete data sets from both initial and follow-up assessment were included in the analyses.

By contrast, in patients, partial correlation analyses revealed a significant correlation between Tu-EL FC and preoperative alertness, indicating that the higher tumor was coupled with the rest of the brain, the worse the attention performance (*r*=.937, *p*=.001). In addition, a trend between Tu-EL FC and postoperative alertness (*r*=.691, *p*=.058) further indicated a potential relevance of the strength of preoperative tumor coupling for postoperative outcome in the attention domain (Fig. 4).

### 3.4 Tu-EL connectivity depending on tumor grading

**Fig. 5:**
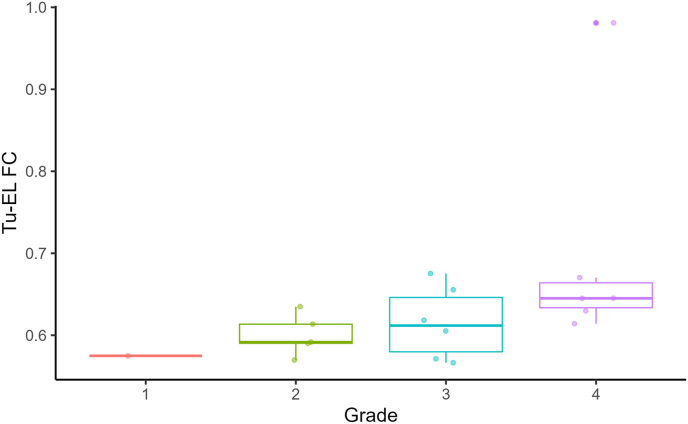
Tu-EL connectivity depending on tumor grading Fig. 5 Plotting the functional connectivity (FC) between tumor and extralesional brain (Tu-EL) in relation to tumor grading revealed increasing mean Tu-EL values with increasing tumor grade, suggesting higher tumor FC of more invasive glioma types.

Plotting Tu-EL connectivity in relation to tumor grading, we found higher mean Tu-EL values, the higher the tumor grade (Fig. 5), which suggests higher tumor FC of more invasive glioma types.

## 4 Discussion

We found resting-state fMRI connectivity of the tumor region to the rest of the brain based on synchronized spontaneous low-frequency BOLD fluctuations to be associated with worse attentional performance, and by trend with worse posttherapeutic attentional outcome in patients. Given the recent evidence of communicating tumor cell networks (Osswald et al., 2015) and their complex interaction with the neural environment (Venkatesh et al., 2015, 2019; Krishna et al., 2023; Yang et al., 2022) with synaptic glutamatergic neuron-tumor signaling promoting tumor-growth (Venkataramani et al., 2019), dysfunctional BOLD coupling of the tumor might reflect such tumor-neural interaction on the macroscale, with impact on cognitive functions.

### 4.1 Cognitive outcome after therapeutic intervention

Complying with previous studies (Habets et al., 2014; Noll et al., 2015; Derks et al., 2019; Cochereau et al., 2016) patients showed preoperatively significant cognitive impairment in all cognitive domains tested. While cognitive performance in our patients tended to be worse at follow-up, deterioration was however not significant at the group level. This observation complies with previous findings, in that cognitive deterioration was reported early postoperatively, but with partial functional recovery within the first six months (Habets et al., 2014; Klein et al., 2012; Noll et al., 2021).

### 4.2 'Cognitive eloquence' from a connectomic perspective

With the increasing understanding of glioma as a systemic rather than focal brain disease, the systemic impact of the tumor on cerebral functions by interaction with large-scale neural networks (Derks et al., 2017, 2021; Hadjiabadi et al., 2018) beyond the tumor region (Derks et al., 2017; Jütten et al., 2021) have repeatedly been reported since. In our study, tumor coupling to the extralesional whole-brain correlated with attentional deficits in patients despite different tumor locations. This complies with previous observations, in that attentional deficits were frequently encountered in glioma patients and unrelated to specific tumor sites or sizes (Hendriks et al., 2018), possibly owing to the more extensive anatomical representations and supramodal character of attentional functions (Raz and Buhle, 2006). While functional neural networks alterations have been associated with cognitive dysfunction (Jütten et al., 2019; Noll et al., 2021; Esposito et al., 2012; Maesawa et al., 2015; Bosma et al., 2009; Kocher et al., 2020), previous findings somehow varied in terms of in- or decreases in FC having been related both to better or worse cognitive performance in glioma patients. Such differences may not only relate to differences in study design or study cohort, but may as well have to be reconsidered in light of the recent revelations of tumor cell networks (Osswald et al., 2015; Yang et al., 2022). Network interaction with the neural environment (Venkatesh et al., 2015, 2019; Krishna et al., 2023; Venkataramani et al., 2019), might indicate that connectomic changes might not only reflect alterations in interneural activity, but could as well relate to tumor-neural interactions.

### 4.3 FC alterations following therapeutic intervention in glioma patients

While we expected cognitive performance to relate not only to connectivity of the tumor, but as well to FC within extralesional areas, mean FC of extralesional brain areas did neither show statistically significant longitudinal alterations, nor associations with cognitive performance in our cohort. This might relate to the fact, that -in view of different tumor locations- global mean rather than network-specific connectivity was addressed here, whereby interregional FC alterations might have been cancelled out.

Post-treatment related cognitive impairment has previously been correlated (Kocher et al., 2020) to reduced MRI resting-state FC within the DMN. Applying graph theoretical analyses on rs-fMRI data, Noll et al. (2021) furthermore observed a better postoperative cognitive outcome to be accompanied by reductions in centrality and resilience connectomic measures, which they suggested as a potential indicator for a postoperative normalization of pathological hyperconnectivity. This could in lack of a control group however not been proven in the current study. Comparing patients to healthy controls in our study, FC within the contralesional hemisphere was significantly increased in patients, providing actual evidence for regional hyperconnectivity in glioma patients even prior to therapeutic intervention. While FC increases might relate to functional reorganization in tumor-distant areas, such hyperconnectivity could alternatively be an expression of pathological neural disinhibition, or even a sign of tumor cell network related activity. Hyperconnectivity thus may not necessarily be functionally beneficial in glioma patients (Derks et al., 2017; Jütten et al., 2019; Sprugnoli et al., 2022), but may reflect different pathophysiological processes with varying behavioral impact, depending on the biological characteristics of the tumor (Derks et al., 2017; Kesler et al., 2017; Stoecklein et al., 2020), and on the time of examination along the disease course (Habets et al., 2014; Noll et al., 2015; Klein et al., 2012). Identifying the true nature and the functional impact of such connectivity alterations is however highly relevant, as it would increase our understanding of tumor-connectomic interaction, and might open the avenue for modulatory (e.g. neurophysiologically, pharmacologically) treatment options.

### 4.4 Functional coupling of the tumor region to extralesional brain areas

Complying with the infiltrative nature of glioma growth and supported by previous IES (Martino et al., 2011) and MEG studies (Tarapore et al., 2012), resting-state FC of the tumor to extratumoral brain regions has previously been related to functionally relevant residual neural activity within the tumor region. Accordingly, previous MEG studies in glioma patients with peri-/eloquent tumor locations showed, that the more the tumor region was preoperatively functionally uncoupled from extralesional brain regions, the less functional impact the tumor resection had on the functional outcome (Tarapore et al., 2012). In our study, albeit only by trend, resting-state fMRI tumor to extralesional brain connectivity was associated with worse postoperative attentional performance outcome which might support this notion. However, tumor BOLD connectivity significantly correlated with worse attentional performance preoperatively, so that such dysfunctional coupling of the tumor to extralesional brain regions might not only reflect interneural communication, but might rather relate to pathological tumor-neural interaction on the macroscale. Accordingly, we found rs-fMRI Tu-EL connectivity to be increased with increasing tumor grade. Such a view is further supported by recent findings correlating tumor BOLD connectivity and global FC alterations with survival in glioma patients (Stoecklein et al., 2020; Sprugnoli et al., 2022). Considering different frequency domains being registered with different methods, MEG and resting-state fMRI based measures of FC therefore might not necessarily relate to the same underlying signal generator registered in the tumor diseased brain (Tarapore et al., 2012; Krishna et al., 2023; Yang et al., 2022; Venkataramani et al., 2019; Bosma et al., 2009), and future studies will have to clarify if and how tumor- and neural network interaction actually interferes with FC as measured by rs-fMRI.

### 4.5 Limitations and perspectives

In view of the small sample size and the heterogeneity of the patient cohort, the present study can only be regarded as exploratory in character. Cognitive outcome predictions will remain difficult, as the posttherapeutic outcome is defined by the sum of multiple factors such as tumor and patients’ characteristics, treatment-related factors, and time of examination. As mean follow-up interval in our study was 4.5 months, cognitive outcome and FC measures at follow-up could have additionally been influenced by neuroplasticity and/or occult tumor progression along the disease course. Moreover, the impact of adjuvant therapies (especially radiotherapy) on cognitive outcome has to be considered as well, which - due to the number of patients in these subgroups - unfortunately could not be statistically adressed in our study. Early postoperative assessment on the other hand might however be confounded by postoperative lesioning and edema and the postoperatively increased physical and emotional distress of the patient. Expecting worse cognitive performance and higher tumor connectivity in patients with more invasive glioma types, IDH wildtype patients tended in fact to show worse attentional performance and higher Tu-EL FC, but with statistical subanalyses of prognostically differing tumor types (based on IDH mutation status) being not feasible in our study due to the limited sample size. In particular, longitudinal attentional performance data in IDH wildtype patients was only incomplete, as these patients were often unable to perform the task, which however in itself is indicative of the worse cognitive performance status of IDH wildtype patients.

In a first exploratory approach, we focused on associations between cognitive performance and FC of the tumor to extralesional whole-brain; future studies with larger patient samples may systematically target specific cognitive networks, which might allow to preoperatively map the 'cognitive eloquence profile' with regard to specific cognitive functions.

Despite the limitations of the present study, it is one of only few studies which longitudinally investigated both, cognitive performance as well as FC with inclusion of a control group, allowing to identify connectivity aberrations compared to normal. Moreover, comprehensive neuropsychological testing in our study longitudinally assessed different cognitive domains rather than correlating FC measures only to basic behavioral outcome parameters. While exploratory in character, our data therefore adds new insights into the cognitive relevance of tumor connectivity, suggesting a connectomic approach for the assessment of cognitive eloquence and outcome estimation, and highlighting the need for further clarification of the pathophysiological correlates of FC alterations in glioma patients in the context of cancer neuroscience.

## 5 Conclusions

Dysfunctional FC between the tumor and the rest of the brain might relate to pathological tumor-neural interaction on the macroscale, reflecting the invasive nature of diffusely infiltrating glioma. Deepening our understanding for connectomic alterations in the context of cancer neuroscience could aid in improving estimation of cognitive impairment and in prognosticating the posttherapeutic cognitive outcome in glioma patients.

## Authorship

Conception and design of the study: CN, VM, KJ, HC.

Acquisition of data: CN, VM, KJ, MW.

Analysis and interpretation of data: CN, VM, KJ.

Drafting the article: CN; VM, KJ.

Revising the article: HC, MW.

Final approval of the version to be submitted: CN, VM, KJ, HC, MW.

## Funding

This research project was supported by a grant from the 10.13039/100005524START Program (AZ141/16) of the 10.13039/501100009398Faculty of Medicine, RWTH Aachen University.

## Declaration of competing interest

The authors declare the following financial interests/personal relationships which may be considered as potential competing interests: Martin Wiesmann reports a relationship with Ab medica, Acandis, 10.13039/100004326Bayer, 10.13039/501100008220Bracco Imaging, Cerenovus, Codman Neurovascular, Dahlhausen, Kaneka Pharmaceuticals, 10.13039/100004374Medtronic, Mentice 10.13039/100024877AB, Microvention, Phenox, 10.13039/501100004830Siemens Healthcare, 10.13039/100008894Stryker Neurovascular that includes: funding grants. Martin Wiesmann reports a relationship with Stryker Neurovascular that includes: consulting or advisory. Martin Wiesmann reports a relationship with 10.13039/501100008220Bracco Imaging, Medtronic, 10.13039/501100004830Siemens Healthcare, 10.13039/100008894Stryker Neurovascular that includes: speaking and lecture fees and travel reimbursement. If there are other authors, they declare that they have no known competing financial interests or personal relationships that could have appeared to influence the work reported in this paper.
